# Drug D, a Diosgenin Derive, Inhibits L-Arginine-Induced Acute Pancreatitis through Meditating GSDMD in the Endoplasmic Reticulum via the TXNIP/HIF-1α Pathway

**DOI:** 10.3390/nu14132591

**Published:** 2022-06-22

**Authors:** Cuicui Zhang, Hai Niu, Chengyu Wan, Xiuxian Yu, Guang Xin, Yuda Zhu, Zeliang Wei, Fan Li, Yilan Wang, Kun Zhang, Shiyi Li, Yuman Dong, Yangying Li, Wen Huang

**Affiliations:** Laboratory of Ethnopharmacology, Tissue-Orientated Property of Chinese Medicine Key Laboratory of Sichuan Province, West China School of Medicine, West China Hospital, Sichuan University, Chengdu 610041, China; 2019324020148@stu.scu.edu.cn (C.Z.); niuhai@scu.edu.cn (H.N.); wanchengyu@wchscu.cn (C.W.); yuxiuxian@wchscu.cn (X.Y.); xinguang@scu.edu.cn (G.X.); zhuyuda@wchscu.cn (Y.Z.); weiran88888@outlook.com (Z.W.); lifan183@outlook.com (F.L.); wangyilanl@outlook.com (Y.W.); zhangk00@outlook.com (K.Z.); lishiyi11@wchscu.cn (S.L.); dongyuman@wchscu.cn (Y.D.); 2019224020170@stu.scu.edu.cn (Y.L.)

**Keywords:** acute pancreatitis, L-arginine, Gasdermin D, endoplasmic reticulum stress, TXNIP

## Abstract

Acute pancreatitis (AP) is one of the most common causes of hospitalization for gastrointestinal diseases, with high morbidity and mortality. Endoplasmic reticulum stress (ERS) and Gasdermin D (GSDMD) mediate AP, but little is known about their mutual influence on AP. Diosgenin has excellent anti-inflammatory and antioxidant effects. This study investigated whether Diosgenin derivative D (Drug D) inhibits L-arginine-induced acute pancreatitis through meditating GSDMD in the endoplasmic reticulum (ER). Our studies were conducted in a mouse model of L-arginine-induced AP as well as in an in vitro model on mouse pancreatic acinar cells. The GSDMD accumulation in ER was found in this study, which caused ERS of acinar cells. GSDMD inhibitor Disulfiram (DSF) notably decreased the expression of GSDMD in ER and TXNIP/HIF-1α signaling. The molecular docking study indicated that there was a potential interaction between Drug D and GSDMD. Our results showed that Drug D significantly inhibited necrosis of acinar cells dose-dependently, and we also found that Drug D alleviated pancreatic necrosis and systemic inflammation by inhibiting the GSDMD accumulation in the ER of acinar cells via the TXNIP/HIF-1α pathway. Furthermore, the level of p-IRE1α (a marker of ERS) was also down-regulated by Drug D in a dose-dependent manner in AP. We also found that Drug D alleviated TXNIP up-regulation and oxidative stress in AP. Moreover, our results revealed that GSDMD^-/-^ mitigated AP by inhibiting TXNIP/HIF-1α. Therefore, Drug D, which is extracted from Dioscorea zingiberensis, may inhibit L-arginine-induced AP by meditating GSDMD in the ER by the TXNIP /HIF-1α pathway.

## 1. Introduction

Acute pancreatitis (AP) is the most common cause of hospitalization for gastrointestinal diseases with extremely high morbidity and mortality [1,2]. L-arginine-induced pancreatitis is one of the most typical and widely used animal models of pancreatitis, which mimics the human disease. Currently, there are no specific treatments for AP. Cleaved Gasdermin D (GSDMD) punches holes in mice acinar cell membrane, resulting in cell death and inflammatory factor release in AP [3,4]. Endoplasmic reticulum stress (ERS) is a key initiating factor in the development of AP [5], which also causes the release of cell contents and inflammatory cascade. Although both ERS and GSDMD up-regulate the release of inflammatory cytokines from acinar cells in AP mice, their effects on the pathogenesis of AP remain unclear.

Thioredoxin-interacting protein (TXNIP) [6,7,8,9,10] is an endogenous thioredoxin negative modulator that plays an important role in retaining the redox balance in cells, which is involved in cell proliferation, differentiation and apoptosis [7]. Thioredoxin is usually a small 12 kDa reductase, which is a common antioxidant [11]. TXNIP inhibits the antioxidant activity of thioredoxin by binding to the protein [7]. TXNIP has been widely studied in other diseases, for example, Bechard et al. found that TXNIP improved pancreatic cancer due to the inhibition of metastasis [12], Zhang et al. found that TXNIP alleviated diabetes by exacerbating oxidative stress [13]. In addition, TXNIP is related to reactive oxygen species (ROS) bursts in cells. A study has shown that the accumulation of GSDMD in the mitochondria increases the release of cytochrome C by increasing the permeability of mitochondria [14], which may lead to the increase of ROS. Whether the gathering of GSDMD in the ER of acinar cells also alleviates AP through the TXNIP signaling pathway is controversial.

Acute lung injury is a common serious complication of AP, which leads to hypoxemia in experimental AP [4,15]. Hypoxia-inducible factor-1α (HIF-1α) ) is a transcription factor that is over-expressed in the presence of hypoxia [15]. Studies have shown that TXNIP regulates diabetic peripheral neuropathy by regulating HIF-1α [15]. Therefore, whether ERS exacerbates the occurrence of oxidative stress through TXNIP and HIF-1α in AP mice is unclear. Therefore, it is necessary to investigate whether the accumulation of GSDMD in the ER of acinar cells regulates ERS and then aggravates oxidative stress through the TXNIP/HIF-1α signaling pathway in AP.

Diosgenin is a kind of natural steroidal sapogenin extracted from Dioscorea zingiberensis, which has excellent anti-inflammatory properties [16,17,18]. A relevant study has shown that dihydrodiosgenin, a diosgenin derive, prevents AP through mitochondrial protection and PI3Kγ/Akt inhibition [19]. However, due to the shortcomings of Diosgenin, such as poor water solubility and low bioavailability, our laboratory carried out a series of structural modification tests on Diosgenin in the later stage and formed Diosgenin derivatives D (Drug D) with higher water solubility and better activity. In our research, we further explore whether Drug D inhibits L-arginine-induced AP through meditating GSDMD in the ER.

ERS and GSDMD mediate AP, but little is known about their mutual influence on AP. Thus, the present study aims to investigate the relationship between ERS and GSDMD in AP. At present, the pathogenesis of AP is unclear, and there is no specific therapeutic drug. Therefore, exploring the pathogenesis of AP and finding AP therapeutic drugs are the research hotspots in this field. So, the objective of this study is to furthermore explore whether Drug D, which is extracted from Dioscorea zingiberensis, inhibits L-arginine-induced AP by down-regulating the expression of GSDMD in ER through TXNIP /HIF-1α pathway.

## 2. Materials and Methods

### 2.1. Reagents and Antibodies

Bicinchoninic acid (BCA) protein assay kit (#MA0082-1) and RIPA lysis buffer (#MA0153) were purchased from Meilunbio (Dalian, China). L-arginine (L-Arg) monohydrochloride (#A6969), Collagenase IV (#C9407), Disulfiram (#HY-B0240) and DAPI (#D9542) were from Sigma-Aldrich (St. Louis, MO, USA) of Merck. GSDMD antibody (#AF4012) was from Affinity (Shanghai, China), REDD1 (Regulated in Development and DNA Damage Responses 1). Antibody (#sc-376671) and p-NF-Kb (p-P65) (#sc-398442) were from Santa Cruz Biotechnology (CA, USA). Resveratrol (#A2122398) was from Aladdin (Shanghai, China). TXNIP(#ab188865) and HIF-1α (#ab1) were from Abcam (Boston, MA, USA). Malondialdehyde (MDA) assay kit (#S0131S), catalase (CAT) (#S0051) assay kit, total glutathione peroxidase (GPX) assay kit with NADPH (#S0058) and anti-β-actin antibody (AF0003) were purchased from Beyotime Biotech (Shanghai, China). Mouse IL-1β ELISA kit (E-EL-M0037c) was purchased from Elabscience Biotechnology Co. Ltd. (Wuhan, China). All other chemicals were purchased from Sigma-Aldrich (St. Louis, MO, USA) of Merck.

### 2.2. Experimental Animal

Healthy wild-type C57BL/6 mice and healthy GSDMD^-/-^ C57BL/6 mice (male, 8 weeks old, weighing 22–25 g) were used to establish the animal model in the experimental study, all of which were separately purchased from the animal feeding center of Sichuan University Health Science Center (Chengdu, China) and Shanghai Model Organisms (Shanghai, China). The total number of mice used in this study is 350, and the number of wild-type mice and GSDMD knockout mice is 305 and 45, respectively. Under specific-pathogen-free (SPF) conditions, all mice were kept in individually ventilated cages with wooden blocks as a bed (5 mice per cage) at 25 °C and 50% humidity at 12 h of dark/light cycle. All mice were kept for 7 days before the experiment. Mice were provided with a standard diet and free tap water. The animal experiment procedure was carried out in accordance with the Guide of Laboratory Animal Care and Use from the United States National Institution of Health and was approved by the Institutional Animal Care and Use Committee (IACUC) of Sichuan University, China (20211363A). In addition, the date of issue of the authorization and the date of expiry for the study is 19 August 2019 and 31 April 2022.

### 2.3. Experimental Model of AP

AP models were induced by L-Arg as described previously with minor modification [20] because a previous study proved that the i.p. injection of L-arginine doses (3 × 3 or 4 × 2.5 g/kg, 10%) were good for inducing AP [21], so, in our study, each mouse was injected with L-arginine at a dose of 3.0 g/kg body/time to build an AP model [20]. The L-Arg solution was prepared in 0.85% saline and filtered to sterilize it with a final concentration of L-arginine to 14%. The mice in vivo were randomly divided into groups (*n* = 7 in each group): (1) control group (CON), (2) AP group (3.0 g/kg body/time, q1 h, 3 h) (MOD) and (3) L-Arg + treatment group (DSF: 100 mg/kg, intraperitoneally, 1 time/day, 3 times; RES: 80 mg/kg, oral, 2 time/day, 6 times; Drug D: 5/10 mg/kg, intraperitoneally, 3 time/day, 9 times). The CON and MOD groups were injected with the same volume of 0.85% saline. The mice were kept on the heating pad between injections and 4 h after the last injection. The mice were euthanized 72 h after the first injection of L-Arg. All experiments were repeated at least 3 times.

### 2.4. Pancreatic Primary Acinar Cells Isolation

Mice pancreatic primary acinar cells were prepared by enzymatic digestion with collagenase IV as described previously [22]. In short, pancreases from mice were taken out and digested with collagenase IV (200 U·mL^−1^) at 37 °C for 19 min. After incubating with Collagenase IV, the separated cells were mechanically broken and filtered through a 100 μm cell strainer and then centrifuged at 700 rpm for 2 min to obtain cell pellets. The cells were re-suspended in N’-a-hydroxythylpiperazine-N’- ethanesulfanic acid hydrogen peroxide (HEPES) solution and stored at 4 °C for later use. The components [23] of HEPES are: 140 mM NaCl, 4.7 mM KCl, 1.13 mM MgCl_2_, 1.2 mM CaCl_2_, 10 mM 4-[2-hydroxyethyl]-1-piperazineethanesulfonic acid [HEPES] and 10 mM D-glucose at pH 7.35–7.45. The cells were then treated at room temperature and used within 4 h after separation.Mice pancreatic primary acinar cells were randomly divided into groups (n = 5 in each group): (1) Control group (CON), (2) AP group (60 mM) (MOD) and (3) L-Arg + treatment group (DSF: 2.5 μM, 50 min; Diosgenin: 50, 100, 200 μM, 50 min; Drug D: 50, 100, 200 μM, 50 min). The CON and MOD group were injected with the same volume of HEPES. DSF was initially dissolved in absolute DMSO and then diluted in HEPES to a final concentration of 1‰ DMSO. Diosgenin and Drug D were initially dissolved in saline and absolute DMSO and kept the final concentration of DMSO at 1‰.

### 2.5. Endoplasmic Reticulum Protein Extraction

ER proteins were prepared using the ER extraction kit (SIGMA, ER0100) according to the manufacturer’s instructions.

### 2.6. Histopathological Examination

Paraffin sections (5 μm) of pancreas tissues were stained with hematoxylin and eosin (H&E). The pancreatic histopathology score was blindly assessed by two pathologists for edema, inflammatory cell infiltration and necrosis, from 0 to 3 (Table 1), assessed as previously described [24,25]. Each slide was observed under an optical microscope (ZEISS, Jena, GMBH).

### 2.7. Immunofluorescence Staining

Briefly, we prepared a paraffin section (3 μm) of the pancreas and dissolved blank goat serum in PBST containing 0.05% Tween 20 to make the concentration reach 5%, then blocked it for 0.5 h. Slices were then incubated with anti-GSDMD (1:100 dilution) and anti-CALNEXIN (1:100 dilution) antibodies overnight at 4 °C at the same time. Then, slices were incubated with corresponding secondary antibodies (1 h, 37 °C) in the dark. After washing, DAPI (4′,6-diamidino-2-phenylindole) (1:2000 dilution) was used for nuclear staining (10 min, 37 °C) in the dark. Stained specimens were visualized with a fluorescence microscope (LEICA LAS X, Wetzlar, Germany). The staining of TXNIP (1:100), HIF-1α (1:100), REDD1 (1:100) and p-IRE-1α (1:300) was as described above.

Isolated primary acinar cells were incubated with L-Arg for 50 min, then fixed with 4% paraformaldehyde for 1 h and incubated with anti-GSDMD (1:100 dilution) and anti-CALNEXIN (1:100 dilution) antibodies overnight at 4 °C in 24-well-plate. Then, cells were incubated with corresponding secondary antibodies (1 h, 37 °C) in the dark. They were then counterstained with DAPI for 10 min. The results were shown with a fluorescence microscope (LEICA LAS X, Wetzlar, Germany).

Pearson’s correlation coefficient values range from 1 for two images whose fluorescence intensities are perfectly, linearly related to −1 for two images whose fluorescence intensities are perfectly, but inversely, related to one another [26]. Thus, we used Pearson’s correlation coefficient to explore the relationship between protein GSDMD and endoplasmic reticulum marker CALENXIN in our article.

### 2.8. Necrotic Cell Death Measurement

Fresh primary acinar cells were treated with L-Arg (60 mM) and incubated at room temperature for 50 min with or without various concentrations of Drug D (50 μM, 100 μM and 200 μM). Then, Hoechst 33342 (50 μg/mL) and propidium iodide (PI, 1 μmol/mL) were used to stain total nuclei and necrotic primary acinar cells characterized by plasma membrane rupture [27], respectively. An automatic ZEISS AX10 imager A2/AX10 cam HRC (Jena GmbH, Germany) was used to record the images. The total number of acinar cells showing PI uptake was calculated from each condition, with a minimum of 1000 cells counted, to provide the percentage (necrosis %) with five isolates per condition.

### 2.9. Oxidative Stress Measurement

According to the instructions, the activities of glutathione (GSH), Catalase (CAT), Glutathione peroxidase (GPX) and malondialdehyde (MDA) in pancreas tissues were detected by a commercial biochemical kit (built in Nanjing, China). The results were shown with a fluorescence microscope (LEICA LAS X, Wetzlar, Germany) and select a representative field of view for the application.

### 2.10. IL-1β Level Measurement

According to the instructions, the level of IL-1β in pancreatic tissues was detected by a commercial biochemical kit.

### 2.11. Western Blot Analysis

Protein lysates were prepared from pancreatic tissues or isolated primary acinar cells by homogenizing in RIPA buffer containing protease and phosphatase inhibitors. Twenty micrograms of protein lysate were loaded on a 12% polyacrylamide gel. Primary antibodies TXNIP (1:1000), REDD1 (1:1000), p-P65 (1:1000), p-IRE1-α (1:1000) and β-actin (1:1000) were used. The image was detected by an enhanced chemiluminescence (ECL) detection system (Millipore, Massachusetts, USA). β-actin was used as a loading control. Data were collected and analyzed from 3 independent samples.

### 2.12. Serum Amylase, Amylase and LDH Secretion Measurement

Blood samples were collected and centrifuged at 3000 rpm for 10 min, and 50 μL of serum were diluted to 200 μL. Serum lipase, amylase and LDH were measured by an automatic biochemical analyzer (Roche, Mannheim, Germany) according to the product specification.

### 2.13. Molecular Docking Studies

In silico investigation was carried out to rationalize the binding mechanism of Drug D with GSDMD (PDB ID: 6N9N). The chemical composition and protein crystal structure of Drug D and GSDMD were imported into Discovery Studio 2019 software. The docking process was conducted using the docking optimization CDOCKER tool in the software.

### 2.14. Statistical Analysis

The data and statistical analysis are in line with the recommendations of pharmacological experiment design and analysis [24,28,29]. Data are expressed as mean ± SEM and analyzed using one-way analysis of variance (ANOVA) or Student t-test. GraphPad Prism version 5.01 (GraphPad Prism Software Inc., San Diego, CA, USA) was used for statistical analyses and preparation of figures. A value of *p* < 0.05 was considered statistically significant.

## 3. Results

### 3.1. GSDMD in Endoplasmic Reticulum of Acinar Cells Is Up-Regulated in L-Arginine-Acute Pancreatitis

**Figure 1:**
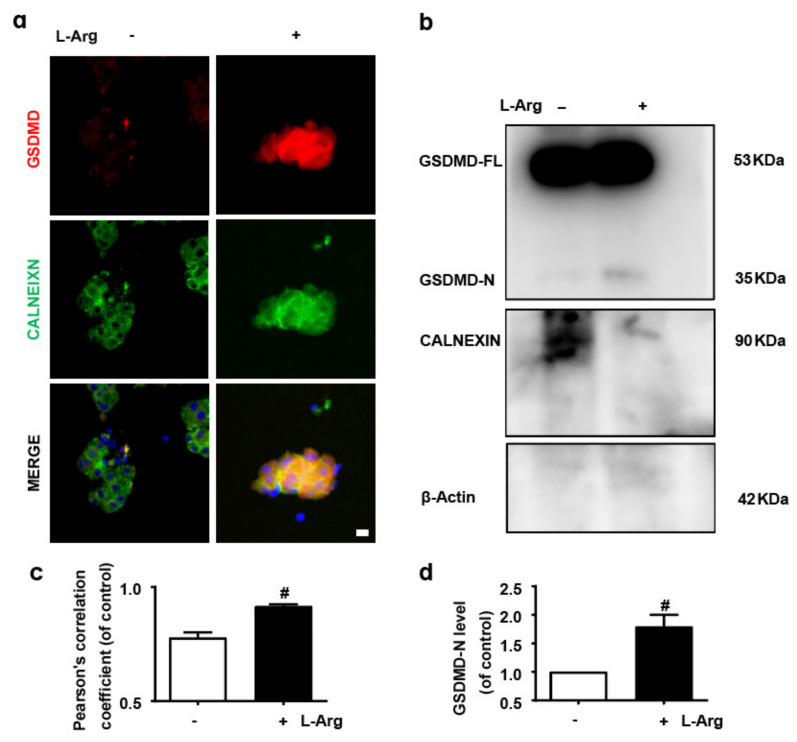
L-arginine induces the accumulation of GSDMD in endoplasmic reticulum of acinar cells. (**a**,**c**) Representative immunofluorescence staining of GSDMD (red), CALNEXIN (green) and DAPI (dark blue) in acinar cells (*n* = 3). Scale bars, 100 μm. (**b**,**d**) The expressions of GSDMD, CALNEXIN and β-Actin in endoplasmic reticulum were analyzed by Western blot (*n* = 3). All values were shown as the mean ± SEM, ^#^ *p* < 0.05 compared with control group. Unpaired Student’s t-tests were used in (**c**,**d**).

The necrosis of acinar cells, the functional unit of the exocrine pancreas, is an important event in AP [1]. Although both ERS and GSDMD up-regulate the release of inflammatory cytokines from acinar cells in AP mice [3,4,5], their effects on the pathogenesis of AP remain unclear. Firstly, this study performed immunofluorescence staining on GSDMD and ER marker protein CALNEXIN [30] to explore the relationship between GSDMD and ER. The results showed that L-Arg up-regulated the accumulation of GSDMD in the ER of acinar cells (Figure 1a,c).

Then, the ER protein of mice’s primary acinar cells was extracted. Firstly, the purity of the isolated ER was confirmed using β-Actin and the ER marker protein CALNEXIN (Figure 1b). Clearly, β-Actin was barely visible. Secondly, we explored the level of GSDMD in the ER by immunoblotting and found that L-Arg increased the level of GSDMD in the ER of acinar cells (Figure 1b,d). We conclude that L-Arg induces the accumulation of GSDMD in ER of acinar cells.

### 3.2. Inhibition of GSDMD Accumulation in Endoplasmic Reticulum Ameliorates Acute Pancreatitis with the Down-Regulation of TXNIP

**Figure 2:**
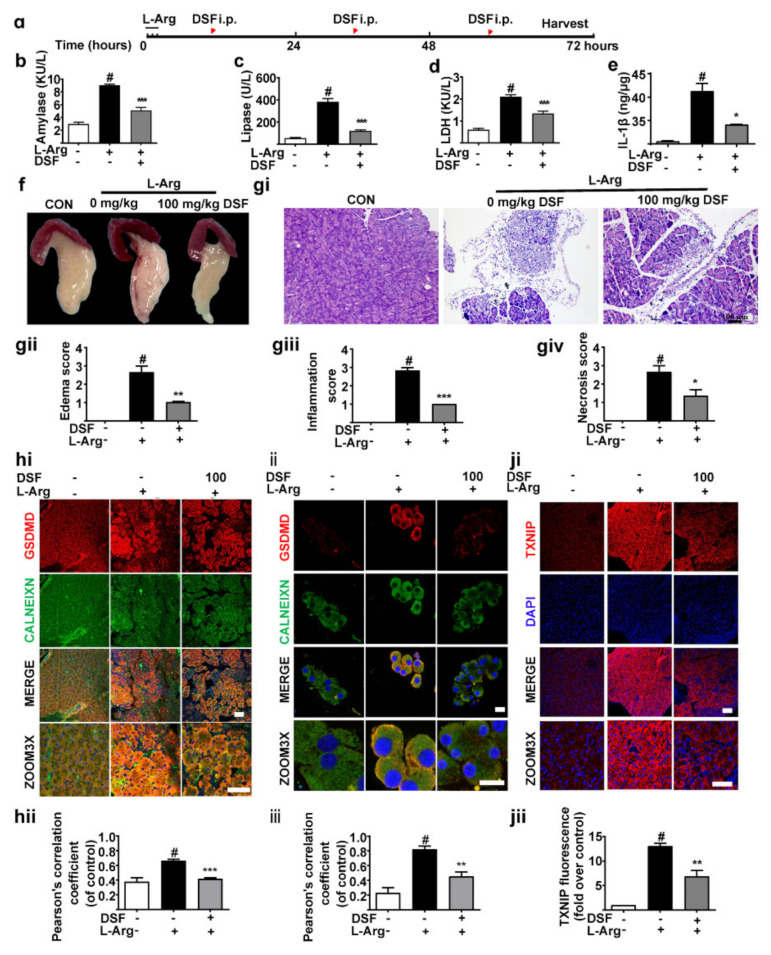
Inhibition of GSDMD accumulation in endoplasmic reticulum ameliorates acute pancreatitis with the down-regulation of TXNIP. (**a**) The experimental schedule. (**b**–**d**) The levels of amylase (**b**), lipase (**c**) and LDH (**d**) in serum were detected (*n* = 3). (**e**) The levels of IL-1β in pancreas tissues were detected (*n* = 3). (**f**) The general morphology of the pancreas was observed. (**gi**–**giv**) Representative H&E images and histopathological scores of the pancreas in L-arginine-acute pancreatitis (*n* = 5). Scale bars, 100 μm. (**hi**,**hii**) Representative immunofluorescence staining of GSDMD (red), CALNEXIN (green) and DAPI (dark blue) in formalin-fixed paraffin-embedded of pancreas tissues and pearson’s correlation coefficient of GSDMD and CALNEXIN (*n* = 5). Scale bars, 100 μm. (**ii**,**iii**) Representative immunofluorescence staining of GSDMD (red), CALNEXIN (green) and DAPI (dark blue) in isolated primary acinar cells from mice and pearson’s correlation coefficient of GSDMD and CALNEXIN (*n* = 5). Scale bars, 100 μm. (**ji**,**jii**) Representative immunofluorescence staining of TXNIP (red) and DAPI (dark blue) in formalin-fixed paraffin-embedded of pancreas tissues (*n* = 5). Scale bars, 100 μm. All values were shown as the mean ± SEM. ^#^ *p* < 0.05 compared with control group, * p < 0.05, ** p < 0.01, *** p < 0.001 compared with mice acute pancreatitis model groups. Unpaired Student’s *t*-tests were used in (**b**–**e**,**gii**–**giv**,**hii**,**iii**,**jii**).

To further evaluate the effect of GSDMD in ER in AP, we employed AP models (Figure 2a). The levels of serum amylase (Figure 2b), lipase (Figure 2c) and LDH (Figure 2d) were significantly increased by L-Arg treatment, accompanied by elevation of pancreas IL-1β (Figure 2e). As illustrated in Figure 2f–giv, L-Arg induced remarkable pathological changes by edema and increased the infiltration of inflammatory cells. Additionally, DSF [31] significantly attenuated pancreatic damage. As we expected, our results also revealed that the expression of GSDMD was significantly increased in ER in pancreas tissues (Figure 2hi,hii) and primary acinar cells (Figure 2ii,iii). Meanwhile, DSF significantly down-regulated the level of GSDMD in the ER (Figure 2hi–iii).

TXNIP is related to ROS bursts in cells, which meditates ERS [14,32]. A study revealed that the accumulation of GSDMD in the mitochondria increases cytochrome C release by increasing mitochondrial permeability [14], which may lead to an increase in ROS. Interestingly, we found that L-Arg increased TXNIP expression remarkably (Figure 2ji,jii). Additionally, DSF treatment decreased the expression of TXNIP (Figure 2ji,jii). Therefore, these results demonstrate that the inhibition of GSDMD accumulation in the ER ameliorates AP with the down-regulation of TXNIP.

### 3.3. TXNIP Inhibitor (Resveratrol) Alleviates Pancreatic Necrosis, Systemic Inflammation and Oxidative Stress in L-Arginine Induced Acute Pancreatitis, without Changes of GSDMD

**Figure 3:**
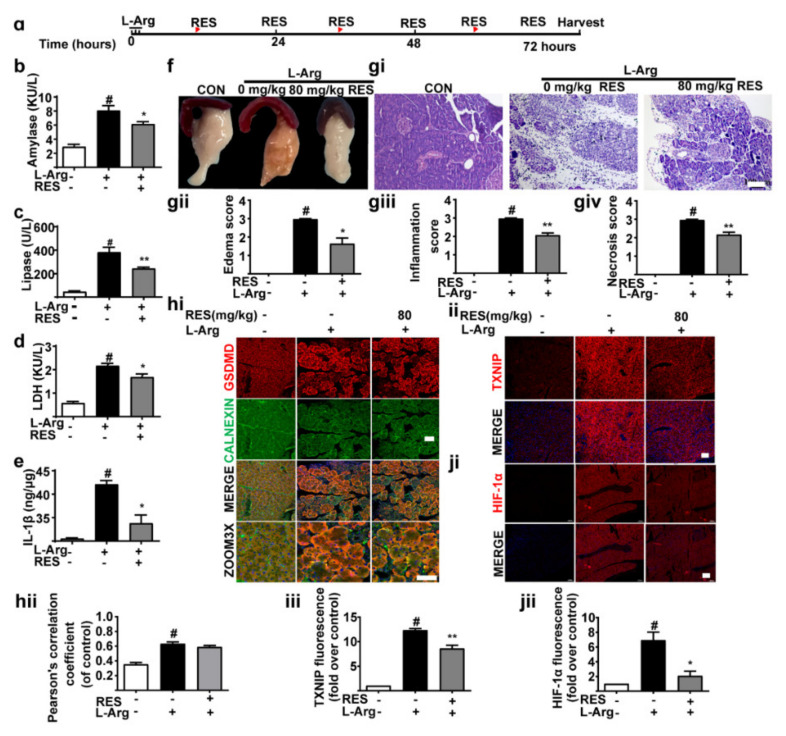
TXNIP inhibitor (Resveratrol) alleviates pancreatic necrosis, systemic inflammation and oxidative stress in L-arginine-induced acute pancreatitis without changes in GSDMD. (**a**) The experimental schedule. (**b**–**d**) The levels of amylase (**b**), lipase (**c**) and LDH (**d**) in serum were detected (*n* = 3). (**e**) The levels of IL-1β in pancreas tissues were detected (*n* = 3). (**f**) The general morphology of the pancreas was observed. (**gi**–**giv**) Representative H&E images and histopathological scores of the pancreas in L-arginine-acute pancreatitis (*n* = 5). Scale bars, 100 μm. (**hi**,**hii**) Representative immunofluorescence staining of GSDMD (red), CALNEXIN (green) and DAPI (dark blue) in formalin-fixed paraffin-embedded of pancreas tissues and pearson’s correlation coefficient of GSDMD and CALNEXIN (*n* = 5). Scale bars, 100 μm. (**ii**,**iii**,**ji**,**jii**) Representative immunofluorescence staining of TXNIP, HIF-1α (red) and DAPI (dark blue) in formalin-fixed paraffin-embedded of pancreas tissues (*n* = 5). Scale bars, 100 μm. All values were shown as the mean ± SEM. *^#^ p* < 0.05 compared with control group, * p < 0.05, ** p < 0.01 compared with mice acute pancreatitis model groups. Unpaired Student’s *t*-tests were used in (**b**–**e**,**gii**–**giv**,**hii**,**iii**,**jii**). Resveratrol represents RES.

In order to explore whether TXNIP signal regulated AP, we used resveratrol (RES) (an inhibitor of TXNIP) in mice AP (Figure 3a). The data revealed that serum amylase (Figure 3b), lipase (Figure 3c) and LDH (Figure 3d) were decreased notably after RES was administered, accompanied by down-regulation of pancreas IL-1β (Figure 3e). As illustrated in Figure 3f–giv, DSF significantly attenuated pancreatic damage. The literature shows that TXNIP regulates the expression of HIF-1α [30]. As shown in Figure 3ii–jii, RES significantly inhibited the expression of TXNIP and HIF-1α. As expected, it did not affect the expression of GSDMD in the ER (Figure 3hi,hii). We conclude that RES mitigates pancreatic necrosis, systemic inflammation and oxidative stress in L-Arg-induced AP via the TXNIP/HIF-1α signal pathway.

### 3.4. Drug D, Targeting GSDMD, Protects against L-Arginine-Induced Necrosis of Primary Acinar Cells

**Figure 4:**
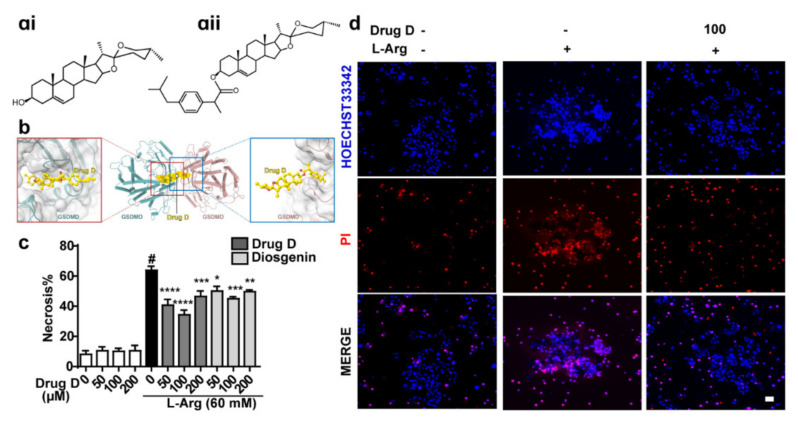
Drug D, targeting GSDMD, protects against L-arginine-induced necrotic cell death pathway activation in isolated primary mice acinar cells. (**a**) The chemical structure of Diosgenin (**ai**) and Drug D (**aii**). (**b**) Scheme of molecular docking. The S-nitrosylation site of GSDMD (C140) is boxed. (**c,d**) The quantification of primary acinar cell necrosis treated with L-arginine (60 mM) and Drug D/Diosgenin at indicated dose and representative image of Hoechst 33342 (blue) and PI (red) staining of primary acinar cells. Scale bars, 100 μm. All values were shown as the mean ± SEM (*n* = 5). ^#^ *p* < 0.05 compared with control group, * p < 0.05, ** p < 0.01, *** p < 0.001, **** p < 0.0001 compared with mice acute pancreatitis model groups. ANOVA was used in (**c**).

Preliminary research in this laboratory found that Diosgenin (Figure 4ai) derivatives had anti-inflammatory and anti-oxidative stress effects [16,17,18]. Here, we tried to explore whether Drug D (one of Diosgenin derivatives) (Figure 4aii) acted as a GSDMD antagonist to protect AP. Results revealed that Drug D potentially interacted with GSDMD (LEU 23, LYS 52 and ILE 467) (Figure 4b), indicating that Drug D is a GSDMD antagonist. Next, we detected whether Drug D decreased the necrosis of acinar cells and found that Drug D protected acinar cells from L-Arg damage obviously (Figure 4c,d). Collectively, Drug D, targeting GSDMD, protects against L-Arg-induced necrosis of acinar cells in vitro.

### 3.5. Drug D Mitigates Pancreatic Necrosis and Systemic Inflammation with the GSDMD Down-Accumulation of Endoplasmic Reticulum in Acute Pancreatitis

**Figure 5:**
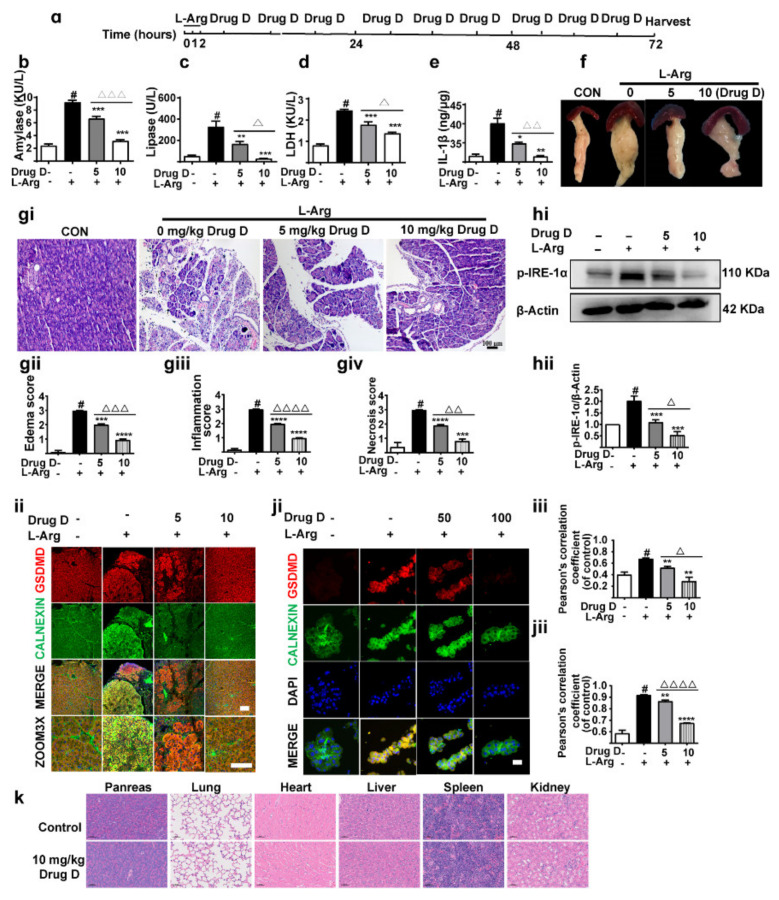
Drug D mitigates pancreatic necrosis and systemic inflammation with GSDMD down-accumulation of endoplasmic reticulum in acute pancreatitis. (**a**) The experimental schedule. (**b**–**d**) The levels of amylase (**b**), lipase (**c**) and LDH (**d**) in serum were detected (*n* = 3). (**e**) The levels of IL-1β in pancreas tissues were detected (*n* = 3). (**f**) The general morphology of the pancreas was observed. (**gi**–**giv**) Representative H&E images and histopathological scores of the pancreas in L-arginine-acute pancreatitis (*n* = 5). Scale bars, 100 μm. (**hi**,**hii**) The expressions of p-IRE1α in pancreas tissues were analyzed by Western blot (*n* = 4)**.** (**ii**,**iii**) Representative immunofluorescence staining of GSDMD (red), CALNEXIN (green) and DAPI (dark blue) in formalin-fixed paraffin-embedded of pancreas tissues and pearson’s correlation coefficient of GSDMD and CALNEXIN (*n* = 5). Scale bars, 100 μm. (**ji**,**jii**) Representative immunofluorescence staining of GSDMD (red), CALNEXIN (green) and DAPI (dark blue) in isolated primary acinar cells from mice and pearson’s correlation coefficient of GSDMD and CALNEXIN (*n* = 5). Scale bars, 100 μm. (**k**) The H&E staining of control groups and 10 mg/kg Drug D groups (*n* = 5), Scale bars, 50 μm. All values were shown as the mean ± SEM. ^#^ *p* < 0.05 compared with control group, ^Δ^ *p* < 0.05, ^ΔΔ^ *p* < 0.01, ^ΔΔΔ^ *p* < 0.001, ^ΔΔΔΔ^ *p* < 0.0001 compared with Drug D (5 mg/kg) group, * p < 0.05, ** p < 0.01, *** p < 0.001, **** p < 0.0001, compared with mice acute pancreatitis model groups. Unpaired Student’s *t*-tests were used in (**b**–**e**,**gii**–**giv**,**hii**,**iii**,**jii**).

Then, we further detected whether Drug D protected AP by targeting the GSDMD in the ER in this study. Here, we evaluated the effects of Drug D on pancreas injury (Figure 5a). Serum amylase (Figure 5b), lipase (Figure 5c) and LDH (Figure 5d) were decreased dose-dependently after treatment with Drug D, accompanied by the decreased pancreas IL-1β (Figure 5e). Supportively, the typical pathological changes such as pancreatic tissue oedema, inflammatory cell infiltration and acinar cell necrosis were also alleviated by Drug D (Figure 5f–giv), and there was no toxicity of 10 mg/kg Drug D compared with control groups (Figure 5k). Furthermore, the level of p-IRE1α (a marker of ERS) in pancreas tissues was down-regulated by Drug D in a dose-dependent manner (Figure 5hi,hii), which further confirmed that Drug D alleviated ERS. In our study, we also found that the accumulation of GSDMD in ER was mitigated by Drug D (Figure 5ii–jii). Therefore, these data demonstrate that Drug D protects AP by regulating the accumulation of GSDMD in ER.

### 3.6. Drug D Alleviates TXNIP Up-Regulation and Oxidative Stress in Acute Pancreatitis

**Figure 6:**
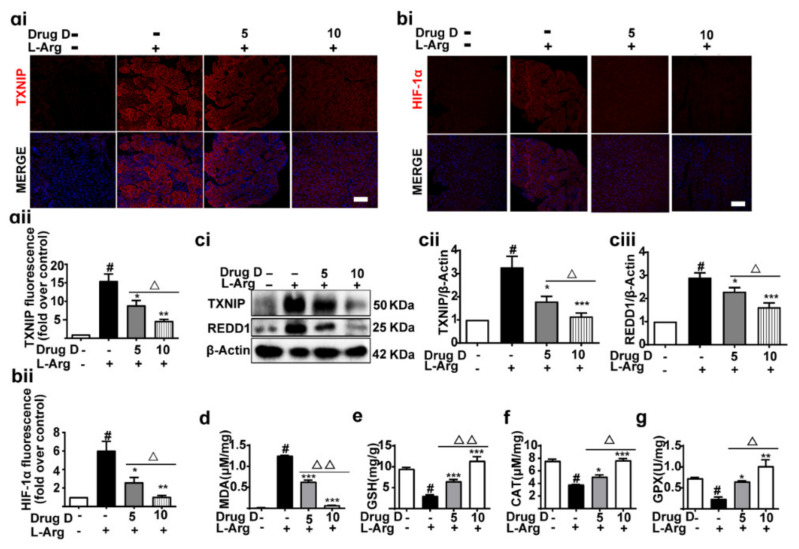
Drug D alleviates TXNIP up-regulation and oxidative stress in acute pancreatitis. (**ai**,**aii**,**bi**,**bii**) Representative immunofluorescence staining of TXNIP (red), HIF-1α (red) and DAPI (dark blue) in formalin-fixed paraffin-embedded of pancreas tissues (*n* = 5). Scale bars, 100 μm. (**ci**–**ciii**) The expressions of TXNIP and REDD1 were analyzed by Western blot in pancreas tissues (*n* = 8). (**d**–**g**) The levels of MDA, GSH, CAT and GPX of pancreas tissues were measured by the corresponding kit (*n* = 3). All values were shown as the mean ± SEM. *^#^ p* < 0.05 compared with control group, ^Δ^ *p* < 0.05, ^ΔΔ^ *p* < 0.01 vs. Drug D (5 mg/kg) group, * p < 0.05, ** p < 0.01, *** p < 0.001 compared with mice acute pancreatitis model groups. Unpaired Student’s t-tests were used in (**aii**,**bii**,**cii**,**ciii**,**d**–**g**).

On the basis of our results, we here speculated that Drug D regulated the severity of AP through TXNIP up-regulation and oxidative stress. Previous studies report that HIF-1α regulates the expression of REDD1, and REDD1 is involved in the balance of oxidative stress [33,34,35]. We found that Drug D down-regulated the expression of TXNIP (Figure 6ai,aii,ci–ciii), HIF-1α (Figure 6bi–ciii) and REDD1 (Figure 6ci–ciii). Additionally, we also found that Drug D decreased the level of MDA (Figure 6d) and increased the levels of GSH (Figure 6e), CAT (Figure 6f) and GPX (Figure 6g) in a concentration-dependent manner. Collectively, these data indicate that Drug D alleviates TXNIP up-regulation and oxidative stress in AP.

### 3.7. Knockout of GSDMD Reduces Drug D-Inhibited Pancreatic Necrosis, Systemic Inflammation and Oxidative Stress in Pancreas Tissues

**Figure 7:**
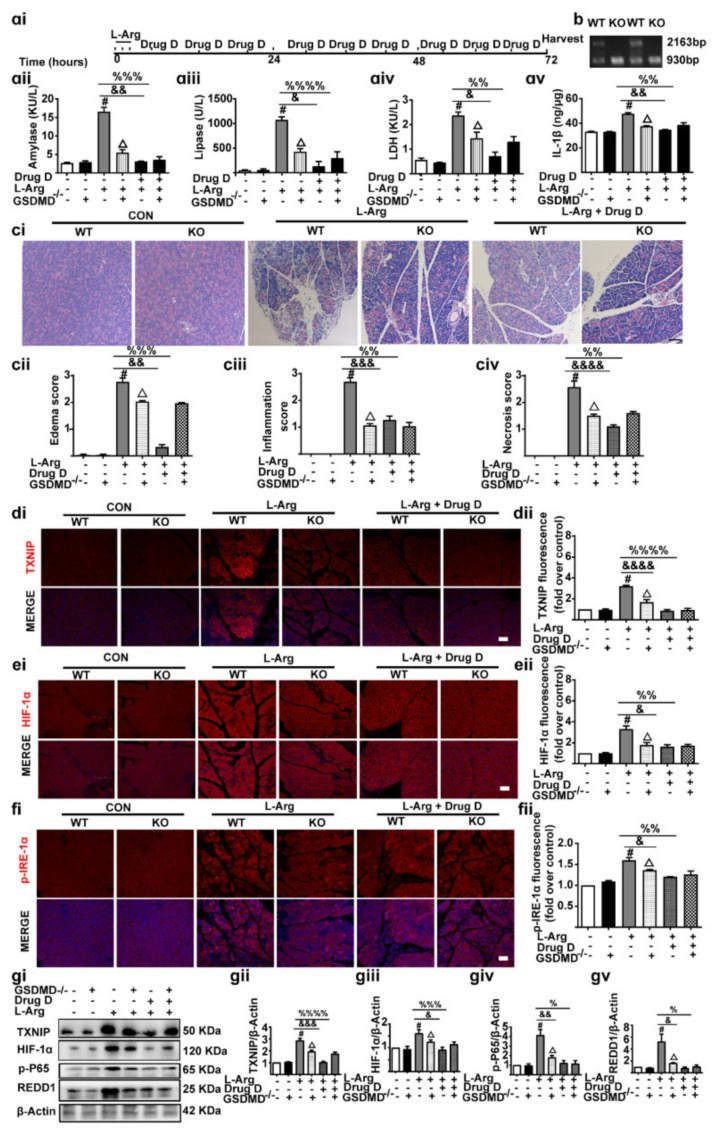
Knockout of GSDMD reduces pancreatic necrosis, systemic inflammation and oxidative stress in acute pancreatitis. (**ai**) The experimental schedule. (**aii**–**aiv**) The levels of amylase (**aii**), lipase (**aiii**) and LDH (**aiv**) in serum were detected (*n* = 5). (**av**) The levels of IL-1β in pancreas tissues were detected (*n* = 5). (**b**) The expression of GSDMD in GSDMD wild-type or GSDMD^-/-^ C57BL/6 mice was detected by PCR. (**ci**–**civ**) Representative H&E images and histopathological scores of the pancreas in L-arginine-acute pancreatitis (*n* = 5). Scale bars, 100 μm. (**di**,**dii**,**ei**,**eii**,**fi**,**fii**) Representative immunofluorescence staining of TXNIP, HIF-1α, p-IRE-1α (red) and DAPI (dark blue) in formalin-fixed paraffin-embedded of mice pancreas tissues (*n* = 5). Scale bars, 100 μm. (**gi**–**gv**) The expressions of TXNIP, HIF-1α, REDD1 and p−P−65 were analyzed by Western blot in pancreas tissues (*n* = 5). All values were shown as the mean ± SEM. *^#^ p* < 0.05 compared with WT mice control group, ^Δ^ *p* < 0.05 vs. GSDMD^-/-^ mice control group, ^&^ *p* < 0.05, ^&&^ *p* < 0.01, ^&&&^ *p* < 0.001, ^&&&&^ *p* < 0.0001 compared with WT mice acute pancreatitis model groups, ^%^ *p* < 0.05, ^%%^ *p* < 0.01, ^%%%^ *p* < 0.001, ^%%%%^ *p* < 0.0001 compared with WT mice acute pancreatitis model groups. Unpaired Student’s t-tests were used in (**aii**–**av**,**cii**–**civ**,**dii**,**eii**,**fii**,**gii**–**gv**). KO represents GSDMD knockout mice, WT represents GSDMD wild type mice.

Our results showed that Drug D ameliorated AP through GSDMD/TXNIP/HIF-1α signaling. Then, we further evaluated the effect of GSDMD^-/-^ (Knockout) on AP (Figure 7ai,b). As we expected, Serum amylase (Figure 7aii), lipase (Figure 7aiii) and LDH (Figure 7aiv) were decreased with GSDMD^-/-^, accompanied by the decreased pancreas IL-1β (Figure 7av). Supportively, GSDMD^-/-^ mice reversed pancreatic tissue oedema, inflammatory cell infiltration and acinar cell necrosis (Figure 7ci–civ). Additionally, we also found that GSDMD^-/-^ mice down-regulated TXNIP/HIF-1α signaling, as shown by the decreased TXNIP (Figure 7di,dii), HIF-1α (Figure 7ei,eii), p-IRE1α (Figure 7fi,fii), REDD1(Figure 7gi–gv) and NF-kB (Figure 7gi–gv). However, the signaling did not further decrease after Drug D treatment. Conclusively, Knockout of GSDMD reduces Drug D-inhibited pancreatic necrosis, systemic inflammation and oxidative stress in pancreas tissues.

## 4. Discussions

AP is an unpredictable and potentially lethal disease [32]. In this study, we demonstrated, for the first time, that GSDMD was overexpressed in the ER of acinar cells, which exacerbated AP by the TXNIP/HIF-1α pathway. Our experimental results also confirmed that Drug D, which acted as a GSDMD antagonist, efficiently decreased ERS by decreasing the accumulation of GSDMD in the ER through the TXNIP/HIF-1α pathway in AP.

Previous studies reported that GSDMD is increased in AP, resulting in the release of proinflammatory cytokines (IL-1β) and then amplifies the local or systemic inflammatory effects [3,4]. Blocking the cascaded amplification of the inflammation improves the outcomes of AP [3,4]. In our study, we also found that GSDMD was significantly up-regulated, which is supported by Gao et al. [3]. Of particular interest, we detected the relationship between GSDMD and ER and found for the first time that GSDMD was accumulated in the ER of acinar cells in AP. This observation might be explained by the fact that ER membranes contain cardiolipins, and the Gasdermin-N domain of GSDMD shows a strong preference for binding to cardiolipin [14]. Together, the accumulation of GSDMD in the ER of acinar cells regulates the severity of AP.

ERS is caused by persistent external damage factors, which are an initiating factor for AP [5]. However, it is unclear whether ERS is associated with GSDMD. Consistent with these studies, our study also found that ERS was obviously up-regulated in AP. In addition, we detected the reasons for ERS and found, for the first time, that ERS was induced by the GSDMD accumulation in the ER. Therefore, inhibiting the accumulation of GSDMD in the ER is crucial to protecting AP.

TXNIP is a regulator of oxidative stress [13] and has been studied in many diseases such as pancreatic cancer [12,36], but how ERS regulates oxidative stress in AP is still unknown. A previous study demonstrated that TXNIP is regulated by p-IRE1α in hepatocytes [37]. Similarly, we also found that TXNIP was up-regulated by ERS. Meanwhile, we used DSF (an inhibitor of GSDMD) to further detect the relationship between ERS and TXNIP. In our study, we found that DSF down-regulated TXNIP, indicating that the accumulation of GSDMD in ER regulates oxidative stress in AP.

Our preliminary studies found that diosgenin and its derivatives had anti-inflammatory and anti-oxidative stress effects [16,17,18]. The effects and mechanisms of Drug D in AP are still unknown. In this present study, it was found that Drug D could bind at the allosteric pocket of GSDMD through molecular docking, suggesting that Drug D might be a GSDMD antagonist. Based on the fact that genetic inactivation (GSDMD^-/-^) and the specific inhibition (DSF) of GSDMD significantly reduce pancreatic necrosis and systemic inflammation in AP [3,4], as expected, we found that GSDMD^-/-^ mice improved AP. However, Drug D did not further improve AP, indicating that Drug D protected AP via targeting GSDMD. Taken together, Drug D is expected to be a potentially effective therapeutic strategy for the development and transformation of new drugs. The limitations of our study are as follows: This study was performed in mice; further studies are needed in humans.

On the one hand, diosgenin and its derivatives also exhibit an inhibitory effect on coagulation and the development of thrombosis [38,39]. On the other hand, it is a close relationship between clotting and the development of inflammation. This relationship is two-sided, coagulation stimulates the development of inflammation, and at the same time, inflammation activates the coagulation cascade [40]. Previous studies have shown that anti-coagulative factors such as heparin [41] and coumarins such as acenocoumarol [42,43,44] and warfarin [45,46] exhibit protective anti-inflammatory and therapeutic effects in acute pancreatitis. These observations suggest that the preventive effect of Drug D in L-arginine-induced acute pancreatitis in in vivo studies is related, at least in part, to the anticoagulative effect of this diosgenin derivative.

A previous study demonstrated that diosgenin is the major metabolite of curcumin steroid saponins in rat serum by HPLC-MS analysis [47]. It provides useful chemical information for the toxicological and pharmacological studies of Dioscorea officinalis. As for the toxicity, oral doses of steroidal saponins up to 562.5 mg/kg in mice did not show any signs of toxicity [47]. However, there were no significant changes in the biochemical and hematological parameters in rats (except for 510 mg/kg/day), and it was concluded that steroidal saponins do not appear to be significantly toxic in their traditional use [47]. In our study, we did not conduct the toxicity test of diosgenin itself, but we verified the toxicity of diosgenin derivative D in vivo. We will discuss and compare the biotoxicity of diosgenin and its derivatives in subsequent experiments.

## 5. Conclusions

In summary, our results show that GSDMD is accumulated in the ER of acinar cells, which aggravates AP in local pancreatic symptoms and systemic inflammation. Drug D, as an inhibitor of GSDMD, improves AP by regulating the GSDMD/TXNIP/HIF-1α pathway. Our research provides new methods for the clinical treatment of AP.

## Figures and Tables

**Table 1:** Histological evaluation of the pancreas.

Parameter	Score	Pancreatitis Pathology Score
Edema	0	no edema
	1	interlobular edema
	2	interlobular and moderate intralobular edema
	3	interlobular edema and severe intralobular edema
Leukocytic infiltration	0	absent
	1	scarce perivascular infiltration
	2	moderate perivascular and scarce diffuse infiltration
	3	abundant diffuse infiltration
Necrosis	0	no necrosis
	1	less than 15% of pancreatic cells involved
	2	15–35% of cells involved
	3	more than 35% of cells involved

## Data Availability

The datasets during the current study are available from the corresponding author on reasonable request.

## Abbreviations

AP: Acute pancreatitis; Drug D: Diosgenin derivative D; DSF: Disulfiram; ER: Endoplasmic reticulum; ERS: Endoplasmic reticulum stress; GSDMD: Gasdermin D; HIF-1α: Hypoxia-inducible factor-1α; L-Arg: L-arginine; ROS: Reactive oxygen species; RES: Resveratrol; TXNIP: Thioredoxin-interacting protein.
